# Erasing memory traces of trauma with eye movement desensitization and reprocessing therapy

**DOI:** 10.3402/ejpt.v7.32545

**Published:** 2016-07-04

**Authors:** Mirjam J. Nijdam, Miranda Olff

**Affiliations:** Department of Psychiatry Academic Medical Center, University of Amsterdam, Amsterdam, The Netherlands, Arq Psychotrauma Expert Group, Diemen, The Netherlands, Email: m.j.nijdam@amc.nl

With its open access character, the *European Journal of Psychotraumatology* aims to promote evidence-based treatments around the world, while at the same time welcoming new forms of treatment without losing its critical scientific eye. Eye movement desensitization and reprocessing therapy (EMDR) is by now a well-established treatment for posttraumatic stress disorder (PTSD). There is good evidence for its efficacy and together with trauma-focused cognitive behavioral therapy (TF-CBT) it is considered to be the first-line treatment for PTSD (Bisson, Roberts, Andrew, Cooper, & Lewis, 2013). All these effective psychotherapies for PTSD have many key elements in common (Schnyder et al., 2015). EMDR is as effective as other forms of trauma-focused psychotherapy (e.g., Nijdam, Gersons, Reitsma, de Jongh, & Olff, 2012) and a recent meta-analysis has shown EMDR even to be slightly superior to TF-CBT for reduction of intrusion and arousal symptoms (Chen, Zhang, Hu, & Liang, 2015). EMDR treatment has also been shown to be efficacious in terms of symptom reduction in refugee populations (Ter Heide, 2011; Acarturk et al., 2016; Mooren, Van de Schoot, de Jongh, & Kleber, 2016) and in survivors of childhood abuse (Ehring et al., 2014). However, some debate still exists about its mechanisms of action (Elofsson, von Schèele, Theorell, & Söndergaard, 2008; Engelhard, 2012; Landin-Romero et al., 2013; Shapiro, 2014).

Clients, clinicians, and researchers who are first introduced to the treatment method EMDR may raise an eyebrow upon hearing about the element of eye movements in this treatment. Surely, at first hearing, it is curious how the therapist will proceed to treat their traumatic memories with the help of eye movements. Regarding the working mechanism of EMDR, most support has been found for the working memory account that states that keeping a traumatic memory in mind and at the same time performing another task results in a competition for working memory resources (Andrade, Kavanagh, & Baddeley, 1997; Gunter & Bodner, 2008). This taxing of the working memory leads to decreased vividness and emotionality of the traumatic images. In this account, eye movements are explained as an essential factor in the efficacy of the treatment by its taxing effect and they may be replaced by various tasks that have a similar effect on working memory (Gunter & Bodner, 2008; Lee & Cuijpers, 2013). When we introduced this special issue, our aim was to identify high-quality studies that present novel findings on EMDR as a treatment method and its working mechanisms. This special issue includes three papers that further investigated the working mechanisms of EMDR in innovative ways, and a paper on a novel application of EMDR, in children with seizure-related anxiety and PTSD symptoms.

With the application of a neuropsychological task of participants’ reading span, Van Schie et al. (2016) investigated the effects of speed of eye movements in relationship to working memory capacity. Based on the working memory account, they hypothesized that participants with smaller working memory capacity would benefit more from slow eye movements, whereas participants with more extended working memory capacity would draw more benefit from fast eye movements during the EMDR procedure. They hypothesized that eye movement speed should be adjusted to someone's working memory capacity for an optimal effect. In testing these propositions in undergraduate students, they found no indication that it would be helpful to adjust the speed of eye movements to the working memory capacity of the participants. Rather, support was found for the fact that the fast eye movements had better effects in both groups in their experiment.

A reaction time task was used in the study by Van Veen et al. (2016) to provide an objective measure of cognitive load of memories that the participants brought in during the EMDR procedure. In their experiment, participants were asked to respond to randomly administered tones while recalling an aversive memory with or without performing eye movements. The authors also included a cognitive effort control condition in which participants recalled another aversive memory while making eye movements. They expected to find that recalling the memory with eye movements would lead to the greatest reduction of working memory load, vividness, and emotionality as compared with the control conditions. Findings in the undergraduate students who participated supported that working memory is indeed taxed by recalling aversive memories according to slowed reaction times on the task. Recalling the aversive memory with eye movements led to a decrease in vividness and emotionality as compared with the other conditions. It also led to a decrease in working memory load but not consistently more than in the control conditions.

Questioning the value of a procedure used in the Dutch EMDR protocol, Matthijssen and Van den Hout (2016) investigated the use of eye movements in the positive closure procedure in 36 clients with PTSD. This procedure is applied at the end of the EMDR session and involves that the client responds to the question what he or she has learned about him- or herself during the session regarding the traumatic event or the theme of the session. Based on the working memory account of EMDR, the authors hypothesized that the positive attribute would be less credible after eye movements as compared to no eye movements. Eye movements proved to have no beneficial effect, but no detrimental effect either. Interestingly, the degree of decrease in Subjective Units of Distress (SUDs) during the session influenced their results; more decline in SUDs during the session increased the credibility of the attribute.

A new application of EMDR therapy was tested by Dautovic and colleagues (2016). The authors applied EMDR in children with seizure-related anxiety or PTSD symptoms who had been referred to a specialized center for epilepsy-related disorders. Due to difficulties in recruitment only five children were included in this study, but the case series report symptom improvement consistent with reliable changes on the instruments used and details the therapeutic changes in every child in important domains. This study provides some preliminary evidence that individuals with seizure-related PTSD and anxiety disorders may respond well to EMDR.

Our hope with this special issue is that it will inspire fellow clinicians and researchers to take the next step in EMDR research in the presented domains and in other areas. The founder of the EMDR treatment, Francine Shapiro, also emphasizes rigorous research to be very important for the credibility of the treatment method and for our understanding of potential new applications of EMDR (e.g., Shapiro, 2014). Novel avenues for efficacy research of EMDR would, for instance, be trauma-related disorders such as addiction and personality disorders. In recent years, short intensive CBT treatments for PTSD have been developed and found to be as effective as standard CBT applied on a weekly basis (e.g., Ehlers et al., 2014), but studies on the effects of intensive EMDR programs and their long-term follow-up are lacking thus far. In conducting these studies, it is important to realize that severe comorbid psychiatric conditions do not preclude patients to benefit from EMDR treatment (Novo et al., 2014; Van den Berg et al., 2015). Although there is some debate on whether all patients are able to immediately receive trauma-focused interventions (Cloitre, 2016; De Jongh & ten Broeke, 2014; De Jongh et al., 2016; Dorrepaal et al., 2014; Ter Heide ea 2016), some patients may clearly benefit from emotion regulation strategies as a means to pave the way for trauma-focused treatments (Cloitre, 2015). The International Society for Traumatic Stress Studies (ISTSS) is currently updating its treatment guidelines for PTSD informed by the research evidence base. But further randomized clinical trial evidence is warranted to provide definitive answers to this question for populations with complex PTSD. We also still know very little about which patients may benefit from EMDR or other interventions (e.g., Nijdam, de Vries, Gersons, & Olff, 2015; Nijdam, Van Amsterdam, Gersons, & Olff, 2015). Future studies on neurobiological predictors and outcomes of EMDR treatment are much needed to understand what happens in the brain and the body during the sessions, with the inclusion of the latest findings on epigenetic, neuroimaging, and neuroendocrinological processes. EMDR is sometimes also applied as a preventive intervention in the direct aftermath of trauma, but randomized controlled trials with credible comparison conditions are not yet available. There is still a lot to be investigated in EMDR treatment research, so this special issue is also a call to expand the evidence base for the benefit of the clients we see on a daily basis.

*Mirjam J. Nijdam* Department of Psychiatry Academic Medical Center University of Amsterdam Amsterdam, The Netherlands Arq Psychotrauma Expert Group Diemen, The Netherlands Email: m.j.nijdam@amc.nl *Miranda Olff* Department of Psychiatry Academic Medical Center University of Amsterdam Amsterdam, The Netherlands Arq Psychotrauma Expert Group Diemen, The Netherlands
